# Estimation of the burden of out-of-hospital traumatic cardiac arrest in Karachi, Pakistan, using a cross-sectional capture-recapture analysis

**DOI:** 10.1186/s12245-020-00283-z

**Published:** 2020-05-14

**Authors:** Minaz Mawani, Iqbal Azam, Muhammad Masood Kadir, Zainab Samad, Junaid Abdul Razzak

**Affiliations:** 1 Department of Epidemiology and Biostatistics, University of Georgia College of Public Health, Athens, GA USA; 2 Department of Community Health Sciences, Aga Khan University, Karachi, Pakistan; 3 Department of Medicine, Aga Khan University, Karachi, Pakistan; 4 School of Medicine, Johns Hopkins University, Baltimore, USA

**Keywords:** Two-sample capture-recapture, Out-of-hospital traumatic cardiac arrest, Burden, Karachi, Pakistan, Emergency care

## Abstract

**Background:**

The burden of trauma-related-out-of-hospital cardiac arrest (OHCA) in developing countries like Pakistan remains largely unexplored due to a lack of organized pre-hospital systems. In order to estimate the burden, we used a two-sample capture-recapture method which has been used in several domains to estimate difficult-to-count populations.

**Methods:**

We obtained 3-month data from two sources: Records of two major EMS (emergency medical services) systems and five major hospitals providing coverage to the city’s population. All adults with traumatic OHCA were included. Information on variables such as name, age, gender, date and time of arrest, cause of arrest, and destination hospital were obtained for these cases and data were compared to obtain a matched sample. Utilizing an equation and different levels of restrictive criteria, estimates were obtained for burden.

**Results:**

The EMS records reported 788 and hospital records reported 344 cases of traumatic OHCA. The capture-recapture analysis estimated the annual traumatic OHCA incidence as 45.7/100,000 (95% CI: 44.2 to 47.3). Estimation of the burden from individual hospital or EMS records underestimated and calculated only 14.6% and 33.9% of the total burden, respectively. Most of the traumatic arrest victims had gunshot wound (GSW) (65.2%) followed by road traffic injuries (RTI) (20.8%).

**Conclusion:**

The actual burden of traumatic OHCA in Pakistan is larger than the burden reported by either the hospitals or EMS services alone. Most of the cases occurred due to GSW and RTI. A multipronged approach is required to manage the problem; from prevention to developing organized trauma care systems and training lay responders in pre-hospital trauma care is vital.

## Background

Among industrialized countries, trauma is the leading cause of death between the ages of one and 44 years [1]. It causes over 5 million deaths each year globally, mostly involving young and productive members of the population [2]. Cardiac arrest secondary to trauma has been reported to have low survival ranging from only 0 to 2.6% and poor functional outcomes for those who survive [3]. Although a culminating event in many, studies have shown that pre-hospital life-saving interventions in out-of-hospital trauma-related cardiac arrest improve survival. Hence, mapping its burden and then instituting systematic interventions is necessary.

In 2013, nearly 1.4 million people died due to road traffic injuries globally and 88% of these occurred in low- and middle-income countries. In the same year, an estimated 118558 deaths due to injuries occurred in Pakistan [4]. In 2010 alone, 28280 and 12580 were estimated to be injured and killed respectively [5]. Yet, no data is available on out of hospital traumatic cardiac arrests from low- and lower-middle-income countries.

In order to estimate the burden of traumatic out-of-hospital cardiac arrest (OHCA) in Karachi, Pakistan, we used a two-sample capture-recapture method which has been used in several domains to estimate difficult-to-count populations.

## Methods

### Setting

This cross-sectional study was conducted in Karachi, the third largest city in the world [6]. Like many other mega-cities, it has a complex health care system comprising of government hospitals mixed with a number of private fee-for-service and charity hospitals. Most hospitals have a provision of providing some level of emergency care though the capacity and quality of care may vary significantly. The detailed methods have been published previously [7].

Briefly, our study sites were five major hospitals and two EMS (emergency medical services) systems. These five comprised of three major government trauma centers of the city that receive majority of trauma visits from all over the city and outside. All of the hospitals were designated teaching hospitals for medical, nursing, and post-graduate training. Generally, trauma patients are transported to the nearest hospital with a designated in-house medico-legal officer due to a prior law mandating an assessment of all trauma patients by the officer before receiving care. Even though the law has since been changed, the practice of bringing patients to these hospitals largely remains [8]. Of the two EMS services in the city, one was primarily a transport service with limited equipment and almost no trained personnel for providing emergency care while the other one is equipped with basic life support equipment and supplies and has either a trained paramedic or a physician onboard. These two are among the three major EMS services of the city and provide coverage to the city’s population [9].

We obtained data from EMS records and prospectively carried out 24/7 surveillance of all patients presenting with out-of-hospital cardiac arrest secondary to trauma to the selected hospitals. We trained 17 data collectors who worked to provide a 24-h coverage in all the five emergency departments and collected information on patients who were brought to the hospital experiencing traumatic cardiac arrest. A study coordinator supervised the data collection to ensure quality. At the end of the shift, all data collectors sent a text message to the study coordinator mentioning the number of eligible patients presenting during their shift. Completed data collection forms were submitted to the study coordinator during the same week. The principal investigator cross-checked the number of forms with the numbers reported via text message to ensure completeness of the records. Cases of traumatic OHCA from EMS were defined as those patients in whom ambulances were dispatched for the sudden cessation of breathing and responsiveness or suspected sudden death due to trauma. Traumatic OHCA from hospital emergency departments was defined as patients presenting to the emergency department with a history of unresponsiveness and sudden cessation of respiration due to trauma and was diagnosed as traumatic cardiac arrest by the treating emergency physician. Patients with other causes of arrests such as cardiac and medical were excluded.

The Ethics Review Committee (ERC) at the Aga Khan University approved the research protocol. In addition, approval from the Institutional Review Boards (IRB) was also obtained from other participating institutions. Written informed consent was obtained from a family member of the patients.

### Data analysis

**Fig. 1:**
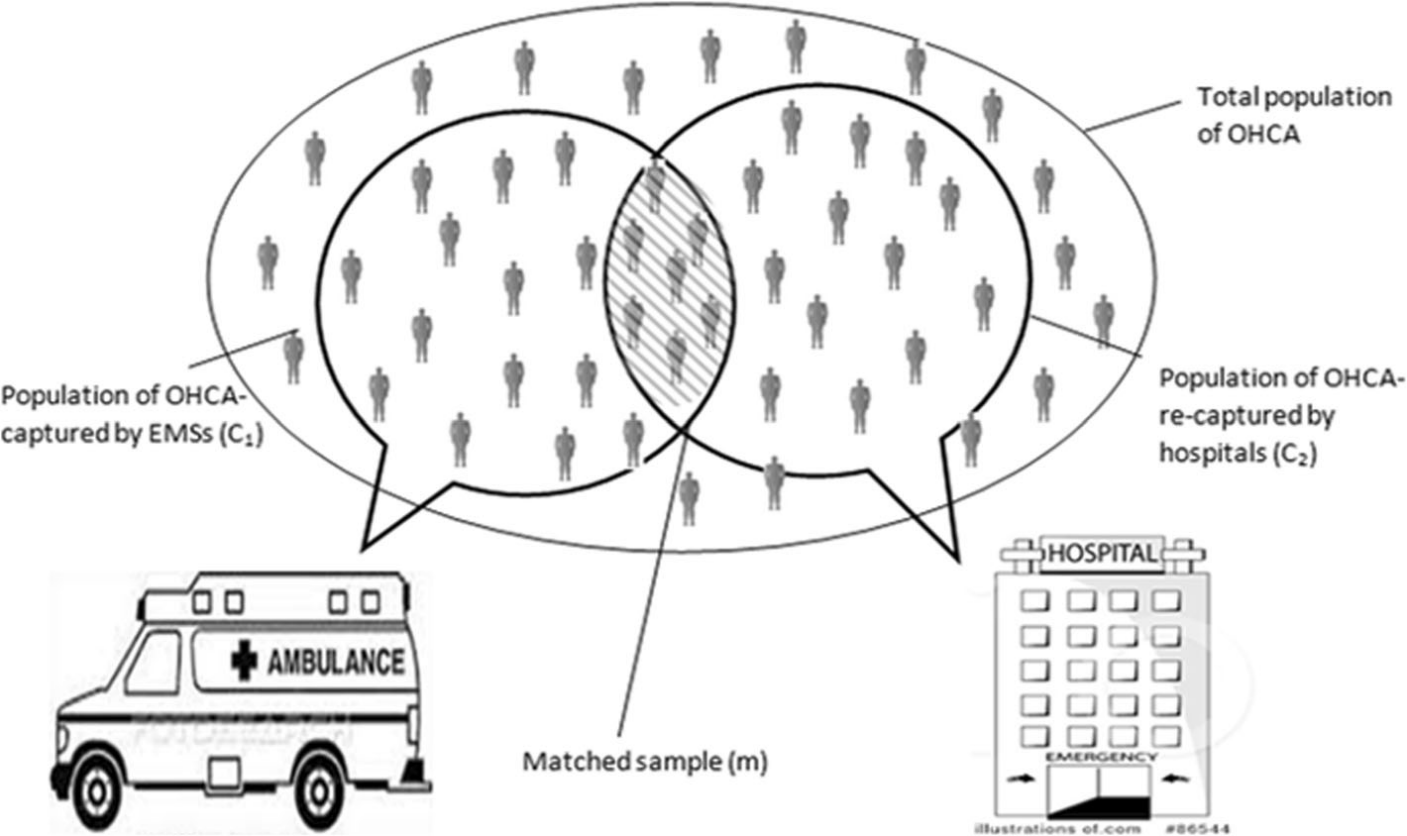
Graphical representation of the capture-recapture application

Data was collected from two sources during January 22, 2013, to April 21, 2013. Capture-recapture methods were used to estimate the burden of traumatic out-of-hospital cardiac arrest. We compared two data sources for identifying cases with common information present in both the lists, also called “matches” [10, 11]. The first capture was all patients ≥ 18 years with traumatic out-of-hospital cardiac arrest from the records of the two major EMS services. The recapture was the similar sample from our hospital-based surveillance system (Fig. 1).

The information on the following variables was recorded: patient’s name, age, gender, date and time of arrest, cause of arrest, and name of destination hospital from both the data sources similar to other published studies [12–14]. These data sources were then compared case by case for identifying matched cases. The degree of matching was defined based on three standards (Table 1). Standard A was the strictest criteria where it was required to have all seven variables match in cases from both the data sources. For each subsequent standard, the criteria were progressively made less strict. However, it was compulsory for all standards to have the same, date, time and gender.

**Table 1:** Description of standards used for identifying matches between EMS and hospital records for traumatic arrest patients

Standard	Compulsory variables	Hospital, cause, patient’s name, and age
A	Date, time, and gender	All four of above same
B	Date, time, and gender	Any three matched
C	Date, time, and gender	Any two matched

In order to consider patient’s names as matched, it was required that both first and last names should be similar in both the sources. The time of arrest was considered to be matched if it was within 1 h. Age was considered to be matched if it was within a 10-year difference. As it is observed that in cases where patient’s age cannot be verified at the time of incident through authentic sources such as a national identification card, then the estimated age is mentioned. Date, gender, cause, and hospital name were required to be exactly the same in both data sources in order to be considered as matched.

The number of traumatic arrest patients with 95% confidence intervals was calculated using the following formulae [15]:

$$ \mathrm{Estimated}\ \mathrm{value}\ \mathrm{of}\ n=\frac{\left(\mathrm{C}1+1\right)\left(\mathrm{C}2+1\right)}{m+1}-1 $$

$$ 95\%\mathrm{confidence}\ \mathrm{interval}=\mathrm{estimated}\ \mathrm{value}\ \mathrm{of}\ n\pm \left(\mathrm{Z}\alpha /2\right)\mathrm{SE}\left(\mathrm{est}.\mathrm{value}\ \mathrm{of}\kern0.28em n\right) $$

$$ \mathrm{Variance}\ \mathrm{of}\ \mathrm{the}\ \mathrm{estimated}\ \mathrm{value}\ \mathrm{of}\;n=\frac{\left(\mathrm{C}1+1\right)\left(\mathrm{C}2+1\right)\left(\mathrm{C}1-m\right)\left(\mathrm{C}2-m\right)}{{\left(m+1\right)}^2\left(m+2\right)} $$

where C1 = number of people in the first capture, i.e., EMS data; C2 = number of people in the second capture, i.e., hospital data and *m* = number of people in both sample (match). This number was divided by 90 to calculate the number per day (from 3 months of data) and multiplied by 365 to calculate annual incidence.

Using Karachi’s population from the last census in 1998 (9.8 million) [16] and a growth rate of 5%, the denominator was calculated to be 20.6 million. The incidence proportion of cardiac arrest patients in Karachi was obtained by dividing the estimated number of cardiac arrest patients by the estimated population of Karachi (*P*^ = *n*/*N*). Analyses were conducted using SPSS (statistical package for social scientists version 19; IBM Corporation, NYC, US).

## Results

During 3 months’ time period (Jan-April 2013), a total of 788 traumatic out-of-hospital cardiac arrest patients were identified from the two major EMS services and 344 from five major hospitals in Karachi. Mean age ± SD was older for the hospital as compared to the EMS group (36.4 ± 12.9 vs. 33.7 ± 10.8, *P* < 0.001) with predominantly male representation (93.2%) in the overall sample. Victims were relatively young with over half (53.7%, *n* = 569) between the ages of 18 and 34 years. Most victims presented with a gunshot wound (65.6%) followed by a road traffic injury (20.9%). A higher percentage of RTAs was found from the hospital sample whereas a higher percentage of the gunshot was found in the EMS sample (*P* < 0.001) (Table 2).

**Table 2:** Characteristics of traumatic arrest patients captured by the hospital and EMS data

Variables	EMS	Hospital	*P* values
	*N* = 788	*N* = 344	
Mean age (SD), range:18–95 years	33.7 (10.8)	36.4 (12.9)	< 0.001
18–34	401 (56.2)	168 (48.8)	0.009
35–44	190 (26.6)	92 (26.7)	
45–64	111 (15.5)	69(20.1)	
65 and above	12 (1.7)	15 (4.4)	
*Missing*			
Gender			0.62
Men	736 (93.5)	319 (92.7)	
Women	51 (6.5)	25 (7.3)	
*Missing*			
Cause of Arrest			< 0.001
Trauma-unknown	12 (1.5)	7 (2.0)	
Road traffic injury	152 (19.5)	84 (24.4)	
Gunshot	528 (67.6)	210 (61.0)	
Fall	8 (1.0)	9 (2.6)	
Wall fell on patient	6 (0.8)	1 (0.3)	
Tortured	26 (3.3)	5 (1.5)	
Blast	34 (4.4)	27 (7.8)	
Suicide	15 (1.9)	1 (0.3)	
*Missing*	7		

*SD* standard deviation, *EMS* emergency medical services

**Fig. 2:**
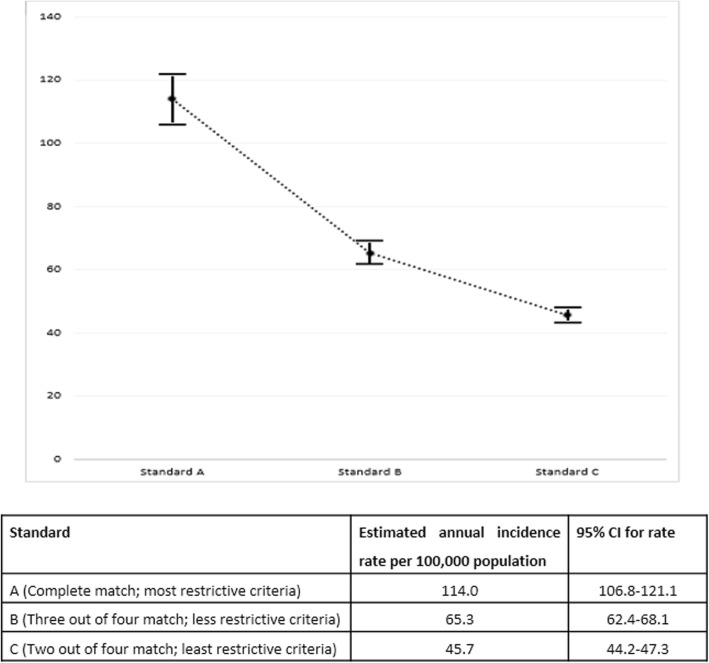
Estimated incidence with 95% confidence intervals of traumatic arrest based on the three criteria

Using the least restrictive criteria (standard c, Table 1), 116 cases matched in both the samples. The number of traumatic arrests for the 3-month period was calculated to be 2325.5 and the number per day was found to be 25.8/day. The annual incidence was calculated to be 9431.3 per year. The incidence rate was calculated by dividing this number by the city’s projected population for the year 2013, i.e., 20.6 million which turned out to be 45.7/100,000 population with 95% CI of 44.2 to 47.3. Using more restrictive criteria resulted in higher estimates (Fig. 2).

Using individual sources of either hospitals or EMS services underestimated the actual burden. In this sample, the burden calculated from individual records of hospital and EMS estimated annual incidence to be 6.77 and 15.5 per 100,000, respectively, which is far lower than the lowest estimate calculated through the capture-recapture method.

## Discussion

To the best of our knowledge, this is the first scientific study exploring the burden of the pre-hospital traumatic arrest. The main findings from this investigation are as follows: (1) Using the capture-recapture method, the annual incidence rate of out-of-hospital traumatic cardiac arrest is calculated to be 45.7/100, 000 which is higher than the burden reported using individual sources. (2) The highest proportion of the traumatic cardiac arrest cases was accounted for by road traffic accidents and gunshot wounds. (3) Majority (80.3%) of out of hospital traumatic cardiac arrest were ≤ 44 years old.

### In our study, the annual incidence rate of out of hospital traumatic cardiac arrest was 45.7/100, 000

Data from Pakistan has so far focused on morbidity and mortality from road traffic injuries [17], fall-related injuries [18], and unintentional injuries, such as poisoning and burns [19], and childhood injuries [4, 20]. However, none of the studies so far have reported the burden of cardiac arrest secondary to trauma in pre-hospital setting [21]. We found that the burden calculated through individual sources of the hospital and EMS records calculated only 14.6% and 33.9% of the actual incidence during the same period. This extent of underestimation is due to the fact that an organized pre-hospital trauma surveillance and care system do not exist and majority of victims are transported to the hospitals by bystanders or family members [8].

In order to address the concern on a policy level and plan interventions, first, there is a need to know population-based estimates. Trauma registries have been established in several developed countries such as Australia [22], Canada [23], France [24], and other developed regions. The capture-recapture method in this scenario was found to be useful in estimating the burden in the absence of a trauma registry. However, setting up a population-based surveillance system for periodic monitoring of outcomes is vital for our population as it provides a systematic approach to measure the impact of trauma, evaluate interventions, inform injury research, and improve public health program planning [22].

One of the reasons for having a huge burden could be that at the time of this study, Karachi had unstable security conditions and the numbers reflect a peak and not a general trend. The security situation in Karachi remains quite variable overtime with peaks reflecting sometimes mass and sometimes targeted violence against some religious and cultural groups. It would be interesting to look at the cause of traumatic arrests when city conditions are stable.

### Road traffic accidents and gunshot wounds accounted for the highest proportion of the traumatic cardiac arrest cases

Driving this number down will require a concerted effort by strict law enforcement including gun control and engaging political and civil organizations. Targeted efforts to prevent road traffic crashes should be taken forth such as legislation to enforce traffic laws, seatbelts, and helmet use for all riders in a systematic fashion [25]. In addition, efforts should be implemented to increase awareness among the general public and sensitization towards the issue.

### Majority (80.3%) of out of hospital traumatic cardiac arrest were ≤ 44 years old

Consistent with published literature from developing countries, our population mostly consisted of individuals in the young and productive age category (18–35 years of age) [25]. Therefore, we should aim to target this population for awareness programs. From the practice perspective, reducing mortality due to these conditions requires a multipronged approach. Efforts should not only focus on significant resource development for trauma care, including the availability of highly trained staff to provide immediate lifesaving care, but also on providing first aid training to laypersons who will be the first responders in a trauma situation majority of the time. In addition, the focus should also be placed on strategies to prevent these incidents in the first place since survival is generally low after a traumatic arrest.

## Limitations

There are four major assumptions for the capture-recapture method to produce consistent results. First, the population should be closed whereby the assumption is that there are no births, deaths, or migrations so that the population size is constant during the time of data collection. In this study, the sampling for capture and recapture took place during a short-time period allowing enough time for the captured individuals to be dispersed and not too long that the population entirely changes which is consistent with the first assumption. Another assumption was that the chances for each individual in the population to be caught should be equal and constant throughout the sampling. This was reflected in our sample as it had a representation from all towns of Karachi since these hospitals and EMS services are the major service providers of the city. The third assumption of this method, that requires independence of the capture sources, is usually difficult to fulfill. Some of the traumatic arrest patients never reach the hospitals. In addition, those patients who access EMS are transported to these major hospitals most of the time. Due to this dependency, our estimates might have underestimated the actual burden. The last assumption is that the capture history for all cases should be accurate. In order to minimize information bias related to inaccurate data, we collected primary data through active surveillance in the hospital. Moreover, the information on the ambulance log is the firsthand information by the ambulance personnel from the site of the event. In addition, since these are medico-legal cases, it is even more carefully recorded by both EMS and hospitals and patients are generally verified through the state-issued identification card and are linked to the hospital records as well.

## Conclusion

The burden of traumatic OHCA calculated using individual records of EMS services or hospitals was underestimated to a great extent. Capture-recapture was found to be a practically useful method in estimating the burden in the absence of surveillance systems. We found that gun violence and road traffic crashes are the major contributors to the burden of traumatic out-of-hospital cardiac arrest in our setting. We suggest targeted efforts to reduce the burden of traumatic arrests through prevention and efforts to improve trauma care systems in Karachi, Pakistan.

## Data Availability

All data generated and analyzed during the current study are available from the corresponding author on a reasonable request.

## Abbreviations

CPR: Cardiopulmonary resuscitation
ED: Emergency department
EMS: Emergency medical services
ERC: Ethics review committee
GSW: Gunshot wound
IRB: Institutional Review Board
OHCA: Out-of-hospital cardiac arrest
RTI: Road traffic injury
SD: Standard deviation
SPSS: Statistical package for social scientists
